# Older parent–adult child contact and proximity across Europe: new evidence, digital communication, and measurement issues

**DOI:** 10.1093/geronb/gbag117

**Published:** 2026-06-27

**Authors:** Bruno Arpino, Marco Tosi, Valeria Bordone

**Affiliations:** Department of Social and Political Sciences, University of Milan, Milano, Italy; Department of Statistical Sciences, University of Padova, Padova, Italy; Department of Sociology, University of Vienna, Vienna, Austria; (Social Sciences Section)

**Keywords:** Intergenerational relationships, Intergenerational solidarity, Comparative family research

## Abstract

**Objectives:**

We aim to provide updated, comparative evidence on the prevalence of frequent (face-to-face, telephone, and digital) contact and close proximity between older parents and their children, and to assess how measurement choices affect cross-national patterns in Europe.

**Methods:**

We use data on 23 European countries from the Survey of Health, Ageing and Retirement in Europe wave 9 (2021–2022) and the European Social Survey round 10 (2020–2022) to estimate the prevalence of frequent contact and close proximity across different approaches: most-contacted versus random child, any versus mode-specific contact, distance versus travel-time thresholds. Cross-national coherence in country rankings is assessed with Spearman rank correlations.

**Results:**

We find a pronounced regional gradient from Southern Europe, with the highest levels of any-mode and face-to-face frequent contact and close proximity, to Nordic and several Continental countries, with the lowest. Eastern Europe lies in-between, with high internal heterogeneity. Instead, digital contact does not follow a clear geographic pattern. Face-to-face and phone contact remain widespread, whereas text and especially video contact are less common. Measurement choices substantially shift prevalence levels, and although country orderings are often similar, concordance is weak for proximity measures based on spatial distance and travel time.

**Discussion:**

We document persistent family-regime differences and interpret digital contact as a supplementary channel of associational solidarity that does not reflect the traditional North/South gradient. We also show that survey design choices matter not only for levels but sometimes also for cross-national orderings.

In contemporary aging societies, family relationships are central to older adults’ wellbeing, providing both support and social control against unhealthy behaviors (Arpino et al., 2023; Connidis & Barnett, 2018; Fingerman et al., 2020; Quashie et al., 2021; Tosi & Grundy, 2019; Umberson & Thomeer, 2020). Within the intergenerational solidarity framework (Bengtson & Roberts, 1991), two dimensions have been largely investigated as facilitators of exchange between older parents and their adult children (Kalmijn & Dykstra, 2006), namely frequency of contact and geographical proximity. In this paper, we contribute to this literature by examining cross-national variations in proximity and contact between older adults and their adult children. In addition to providing updated comparative evidence on the prevalence of frequent contact and close proximity between older parents and their children, we assess how measurement choices affect cross-national patterns in Europe across different data sources and types of contact.

Cross-national analyses in Europe are especially informative, due to a context that combines advanced population aging with marked heterogeneity in family configurations, from “strong family” systems showing short-distance proximity and very frequent contact in Southern Europe to “weak family” systems characterized by longer distances and regular but less intensive contact in Northern Europe (Bordone, 2009; Dykstra & Fokkema, 2011; Hank, 2007; Mönkediek & Bras, 2014; Tomassini et al., 2004). However, much of what we know about parent–child contact and proximity in Europe is largely based on a single data source, i.e., the Survey of Health, Ageing and Retirement in Europe (SHARE) and rests on relatively old studies published in the years following the first SHARE waves (Bordone, 2009; Dykstra & Fokkema, 2011; Fokkema et al., 2008; Hank, 2007; Kohli et al., 2005; Mönkediek & Bras, 2014). Two notable exceptions use International Social Survey Programme (ISSP) data from 1986 and 2001 (Kalmijn & De Vries, 2009; Treas & Gubernskaya, 2012), while a few studies rely on national surveys and compare a limited number of countries (Glaser & Tomassini, 2000; Tomassini et al., 2004). Such an overreliance on a single source is problematic because results might be driven by its specific methodological features. In fact, while SHARE offers rich information on all children, it conflates all forms of contact (face-to-face and remote). More recently, the European Social Survey Round 10 (ESS-10, 2020–2022) collected cross-national data on parent–child contact and proximity, distinguishing face-to-face, phone, and digital (text, video calls) contact types. Studies using ESS-10 confirm the previously found substantial variation across countries (Arpino & Failli, 2024b; Laumert, 2025), but measurement differences between SHARE and ESS-10 raise comparability concerns and call for a methodological reflection.

Against this background, this paper makes three contributions. First, it provides updated estimates of parent–child contact and proximity across 23 European countries, including several Southern and Eastern countries not covered in earlier comparative analyses. Second, it offers a systematic comparative analysis of digital communication alongside face-to-face and telephone contact, considering four forms of contact separately (face-to-face, phone, text, video). Third, it assesses the consequences of different methodological approaches to measuring parent–child contact and proximity in SHARE and ESS-10. Using simple “simulation” exercises, we evaluate how country rankings of frequent contact and geographical closeness respond to choices such as focusing on the most-contacted versus a random child, aggregating versus separating contact modes, and using distance versus travel time for proximity. Our purpose is not to explain cross-national differences through direct tests of macro-level hypotheses. Rather, we provide an updated mapping and methodological assessment for the observed patterns. Nor do we aim to directly compare levels between SHARE and ESS-10, given their design differences. Rather, we aim to gauge how a few crucial choices may contribute to cross-survey differences. Insofar as feasible, we apply ESS-style choices to SHARE data and vice versa to evaluate the resulting shifts in country rankings and the consistency of the North/South gradient found in previous studies. While we focus on parents’ reports, much of the reasoning applies equally to child-based data. Similarly, although this study relies on European data, the methodological discussion is relevant to data collection on parent–child relationships more broadly. In particular, SHARE’s approaches to measuring parent–child contact and proximity closely resemble those used in other major aging studies (e.g., the US Health and Retirement Study and its sister studies), reinforcing the wider applicability of our points across this family of aging surveys. Most of the points discussed below also apply to contact and proximity in relationships other than parent–child ones.

## Background

### Cross-national differences in parent–child coresidence, proximity, and contact across Europe

Comparative research has long documented a persistent European gradient in intergenerational ties: very frequent parent–child contact, short-distance proximity, and co-residence are generally highest in Southern Europe, lowest in Northern Europe, and somewhat intermediate in Central and Eastern Europe (Hank, 2007; Isengard, 2013). These differences can be interpreted through the idea of institutionalized generational interdependence, that is, the extent to which public policies require families to provide welfare or instead enable residential and economic autonomy across generations (Dykstra, 2018; Saraceno & Keck, 2010). In the Nordic countries, more defamilializing policy mixes support earlier home-leaving and reduce the practical need for co-residence or very short-distance living; lower residential intensity should therefore not be read as weaker solidarity overall, but as a more autonomy-supporting form of it (Daatland et al., 2011). By contrast, Southern European countries combine more familialistic welfare arrangements with later and less secure transitions to adulthood and housing systems in which access to independent housing often depends on parental help and homeownership-based transfers; these conditions tend to keep adult children longer in or near the parental home and may attach reciprocal expectations of contact and support to housing assistance (Aassve et al., 2013; Arundel & Ronald, 2016). Comparative studies also suggest that Eastern European countries are not simply “in between” North and South. Rather, several Eastern countries resemble Southern Europe more than Nordic Europe. This pattern has been linked to stronger filial norms, denser practical intergenerational exchanges, and post-socialist housing legacies—mass privatization, weak rental sectors, and “housing welfare by default,” which increase dependence on family resources and often prolong co-residence or near-residence (Heylen et al., 2012; Saraceno & Keck, 2010; Stephens et al., 2015; Tosi & Grundy, 2019). Related comparative research on other forms of intergenerational ties, such as grandparental childcare, points in a similar direction, with several Eastern countries aligning more closely with Mediterranean than Nordic patterns (Bordone et al., 2017; Zanasi et al., 2023). For other Eastern European countries, the opposite holds (Dykstra, 2018; Iacovou & Skew, 2011). In particular, the Baltic states often deviate from patterns observed in other Eastern European countries (Iacovou & Skew, 2011). Because geographic proximity structures opportunities for face-to-face interaction, contexts with delayed residential autonomy also tend to show more frequent in-person contact; however, contact patterns cannot be reduced to distance alone, since phone and other remote forms of contact also reflect normative expectations, practical needs, and the broader organization of intergenerational support (Arpino & Failli, 2024b; Kalmijn & De Vries, 2009).

### Digital contact between older parents and children

From its original formulation, the intergenerational solidarity model has treated both face-to-face interaction and remote communication (traditionally, for example, by landline telephone) as part of the associational solidarity dimension (Bengtson & Roberts, 1991). In our increasingly digitalized world, digital contact has been recognized as crucial for intergenerational solidarity, and the term “digital solidarity” has been proposed (Peng et al., 2018). Although face-to-face interaction remains the main way older parents and adult children share activities and experiences, the increasing use of digital tools is transforming intergenerational communication. Video calls, instant messaging, and social media now complement traditional forms of contact, enabling family members to sustain emotionally close relationships, even despite physical distance (Hwang et al., 2024; Peng et al., 2018; Tosi & Arpino, 2025).

Comparative data suggest strong complementarity between old and new media. Arpino and Failli (2024b) find that frequent texting or video calls among older European parents usually go hand‐in‐hand with more telephone and face‐to‐face contact. Danielsbacka et al. (2023) similarly report reinforcement rather than displacement between digital and traditional communication modes in Finland among various kin ties. Emotional closeness remains a key driver of all contact types, while geographical distance tends to reduce face‐to‐face contact but is partly offset by video/phone calls.

Crucially, digital usage differs by socio-demographics and context. Older age, lower education or income, and rural residence are associated with lower internet adoption and digital literacy. Hunsaker and Hargittai (2018) document a persistent “second-level digital divide” among older adults, and Arpino and Failli (2024a) note that such digital inequality poses barriers to digital contact. Thus, technological factors may matter together with family norms to explain variation in digital parent–child contact. Cross-national variation in digital contact is consistent with the multilevel framework developed by Qian and Hu (2024) who argue that family digitalization must be understood in light of national policy, welfare, and cultural regimes.

### Measuring parent–child contact and proximity in comparative surveys

Frequency of contact and geographical proximity between parents and children may seem straightforward to measure in surveys, but several methodological choices must be made. Below, we primarily focus on the choices made by SHARE and ESS-10, while also referencing other key cross-national and national surveys used in intergenerational research. To facilitate the discussion below, Table 1 summarizes the main measurement approaches to parent–child contact and proximity considered in this study and shows how they are operationalized in SHARE-9 and ESS-10.

**Table 1:** Measurement approaches to parent–child contact and proximity compared in the study: operationalizations based on SHARE-9 and ESS-10.

Reference child	Dimension	Measurement approaches	SHARE-9 measures	ESS-10 measures
**All children**	Contact	Any mode	SHARE most contacted child	
		Mode specific		
	Proximity	Spatial	SHARE closest child	
		Travel time		
**Random**	Contact	Any mode	SHARE contact random child	ESS highest contact type
		Mode specific		ESS face-to-face; ESS text; ESS video; ESS phone
	Proximity	Spatial	SHARE close random child	
		Travel time		ESS close random child

*Note*. SHARE-9 = Survey of Health, Ageing and Retirement in Europe, wave 9 (2021–2022); ESS-10 = the European Social Survey, round 10 (2020–2022). The table summarizes the measurement approaches compared in this study and how they are operationalized in the Survey of Health, Ageing and Retirement in Europe, wave 9 (SHARE-9), and the European Social Survey, round 10 (ESS-10). “SHARE most contacted child,” “SHARE closest child,” and “SHARE contact/close random child” are measures derived from the all-children information available in SHARE-9. “ESS highest contact type” is derived from ESS-10 as the highest frequency reported across face-to-face, phone, text, and video contact for the randomly selected child. In ESS-10, the reference child is selected using the closest-birthday rule. Frequent contact is defined as more than once a week and includes coresidence. Close proximity is defined as living within 25 km in SHARE-9 and within 30 min of travel in ESS-10. Empty cells correspond to measures impossible to estimate with a specific survey.

### Which child(ren) is (are) referenced?

A first key decision in measuring parent–child contact and proximity concerns how many—and which—children are covered (see the first column of Table 1). One option is to ask about each child separately: the *all-children approach*, as used in SHARE (as well as in the Generations and Gender Survey, GGS). Another one is to focus on one randomly drawn child: the *random-child approach*, as done in ESS-10. A third approach selects the closest child(ren)—i.e., the most frequently contacted or most geographically proximate (as in ISSP 1986 and 2001 and in Italy’s Families and Social Subjects, FSS). A fourth is to select the oldest child(ren) (as in the German Ageing Survey, DEAS, and Generational Transmissions in Finland, Gentrans). Note that in the first two waves of SHARE, parent–child relationships were measured in a fifth way, i.e., for up to four children; for respondents with more than four children, a mix of age and geographic proximity rules was applied (see SHARE [2024] for details).

These two approaches speak to two related but distinct quantities: whether parents have frequent contact with at least one child and how frequent contact with a typical child is. The SHARE *all-children approach* enables within-parent analyses of variation in contact/proximity across children. Researchers can assess whether parents have frequent contact/high proximity with some children while maintaining less frequent contact/greater distance with others as well as mixes of them, and examine determinants and consequences of these patterns (e.g., Albertini & Arpino, 2018). This type of analysis is not allowed by the ESS-10 *random-child approach.* Such an approach, however, reduces respondent burden by collecting information on a single child, facilitating the inclusion of parent–child items in general-purpose surveys. While it does not allow within-dyad analyses, the random-child approach can still provide aggregate estimates of parent–child relationships, which are, in principle, representative of the overall average amount of contact that parents have with their children. ESS-10, specifically, implements the “closest birthday” method, asking respondents to report on the child whose birthday most recently preceded the interview. Under such a random selection of the reference child, this design should yield representative population averages of parent–child contact. However, estimates can diverge from an *all-children* average if sibship size is related to per-child contact. For example, if parents with many children have less frequent contact with each child than parents with fewer children, the *all-children* mean will be lower than the random-child mean; cross-national comparisons may therefore be sensitive to differences in fertility.

### How are contact and proximity defined and measured?

A second choice related to the measure of contact is whether to ask about any type of contact in a single item or to separate contact modes (see Table 1). A possible strategy is to aggregate all forms of contact—face-to-face and remote—into a single item (*any mode*; used in SHARE, GGS, and DEAS). An alternative strategy asks separate questions for face-to-face (F2F) and for each distinct form of remote contact (e.g., phone, text, video call, social media)—a *mode-specific* approach is used in ESS-10 as well as in FSS and Gentrans. This enables a more nuanced understanding of parent–child ties and permits analyses of potentially different determinants and effects by type of contact (e.g., Arpino & Failli, 2024a; Arpino et al., 2021; Hwang, 2026; Tosi & Maraniello, 2026). This level of detail is feasible when questions pertain to one (random) child or to a small number of children, given the burden for respondents in general-purpose surveys. An intermediate approach uses two items, distinguishing only between F2F and remote overall contact (e.g., in ISSP).

While answer categories matter for both contact and proximity questions, categories of contact frequency can be usually harmonized to a meaningful common scale (see Methods below for our harmonization of SHARE and ESS-10 in this respect). For proximity instead, the metric is a crucial choice: spatial distance (e.g., kilometers, as in SHARE, FSS, and Gentrans) versus temporal distance (e.g., hours/minutes of travel, as in ESS-10, GGS, and ISSP). Hybrid designs exist as well: DEAS primarily uses spatial categories, but, for children living in different neighborhoods within Germany, it further distinguishes by travel time. Distance in kilometers and travel time are correlated, and, in either metric, greater geographical separation is consistently associated with less frequent contact and exchanges (Artamonova & Syse, 2021; Bordone, 2009; Ermisch & Mulder, 2019; Hank, 2007). However, they can diverge due to terrain, infrastructure, traffic, and transport options. Each metric has advantages: kilometers provide an objective, stable gauge of spatial dispersion, whereas travel time captures functional accessibility that reflects local conditions and typical transport modes (Artamonova & Syse, 2021; Ermisch & Mulder, 2019).

Note that the *most-contacted-child* and *closest-child approaches* will, by construction, overestimate average contact and proximity. Crucially, however, these approaches also answer different substantive questions compared to the random-child one: random-child estimates describe contact or proximity with a typical child, whereas most-contacted or closest-child indicators capture whether parents have frequent contact with, or live close to, at least one child. These latter measures are often considered important for older parents’ health and the probability of receiving support. The distinction between the two approaches also matters for how results are interpreted, including questions about social isolation risk versus the typical intensity of intergenerational ties. In this manuscript, the focus is on the extent to which the different approaches provide similar aggregate patterns of parent–child contact and proximity.

## Data and method

### Data

We use SHARE wave 9 (SHARE-9, 2021–2022) and ESS round 10 (ESS-10, 2020–2022)—the most up-to-date cross-national sources on parent–child relationships available for multiple European countries. ESS covers residential populations aged 15+; SHARE targets adults aged 50+ and their partners, excluding institutionalized or out-of-country individuals. ESS-10 combines face-to-face and mixed-mode designs across countries; SHARE-9 uses face-to-face interviews with an extended fieldwork period.

We harmonize as much as possible the two datasets by selecting parents aged 60+ with at least one child aged 12+ living in the 23 countries included in both surveys: Austria, Belgium, Bulgaria, Croatia, Cyprus, Czech Republic, Estonia, Finland, France, Germany, Greece, Hungary, Italy, Latvia, Lithuania, the Netherlands, Poland, Portugal, Slovakia, Slovenia, Spain, Sweden, and Switzerland. The 60+ threshold avoids representativeness issues for people in their 50s in SHARE-9 where refreshment samples were not added. In SHARE, we use the “family respondent” (reporting on behalf of both partners) as the analytic unit. The child age threshold (12+) reflects ESS routing. The final sample includes 32,340 parents in SHARE-9 and 13,832 in ESS-10.

### Measures

Table 1 provides an overview of the measurement approaches compared in this study and how they are operationalized in SHARE-9 and ESS-10. In order to provide updated cross-national patterns of parent–child contact and proximity from both datasets, we follow previous research (Hank, 2007; Tosi & Grundy, 2019). In SHARE-9, we derive measures for the most contacted child (“SHARE most contacted child”) and the geographically closest child (“SHARE closest child”); in ESS-10, we consider each contact mode separately (e.g., “ESS face-to-face”) and close proximity as living within a 30-min travel radius. To assess survey-specific methodological choices, we then construct additional measures. First, we simulate a random-child design in SHARE (“SHARE contact/close random child”), mirroring ESS-10: we estimate parent–child contact/closeness by selecting one child at random for each SHARE respondent. Second, we simulate an “any mode” measure in ESS-10 by taking the highest frequency among the four modes (“ESS highest contact type”), thus approximating how respondents might answer a single item on any mode. This may still differ from SHARE if SHARE respondents implicitly sum contact frequency across modes (e.g., adding face-to-face and phone contacts) rather than basing their response on the most frequent single mode. However, we minimize the risk that this matters by focusing on frequent contact. Answer categories ranged in both cases from “daily” to “never” but on different scales. In SHARE-9, the original response categories include: daily, several times a week, about once a week, about every two months, about once a month, less than once a month, never. ESS-10 categories include: several times a day, once a day, several times a week, several times a month, once a month, less often, never. Defining frequent contact as more than once a week (similar to e.g., Hank, 2007) guaranteed comparability across the surveys. In the baseline analyses, co-resident dyads are treated as having frequent face-to-face contact, reflecting daily interaction within a shared household. At the same time, co-residence is conceptually distinct from active contact across separate households and may partly reflect national differences in leaving-home behavior. We therefore also performed a robustness check analysis excluding co-resident dyads from the contact measures. For proximity, SHARE provides ordinal distance categories; ESS-10 provides travel time. Following common practice, we define “living close” as within 25 km in SHARE (e.g., Hank, 2007) and within 30 min in ESS (similar to Shelton and Grundy [2000] on other data). We do not attempt direct conversion between kilometers and travel time.

All estimates are weighted using post-stratification and design weights that account for sampling probabilities, nonresponse, and non-coverage (see Bergmann et al. [2024] for SHARE-9; see ESS [2020] for ESS-10). We also calculate concordance among the country rankings obtained with different measures. Specifically, we report the Spearman correlation coefficient (*ρ*) for pairwise correlations, which ranges between −1 and +1, and it is interpreted as the correlation among the rankings of the countries according to a pair of measures.

## Results

### Frequent contact

**Figure 1:**
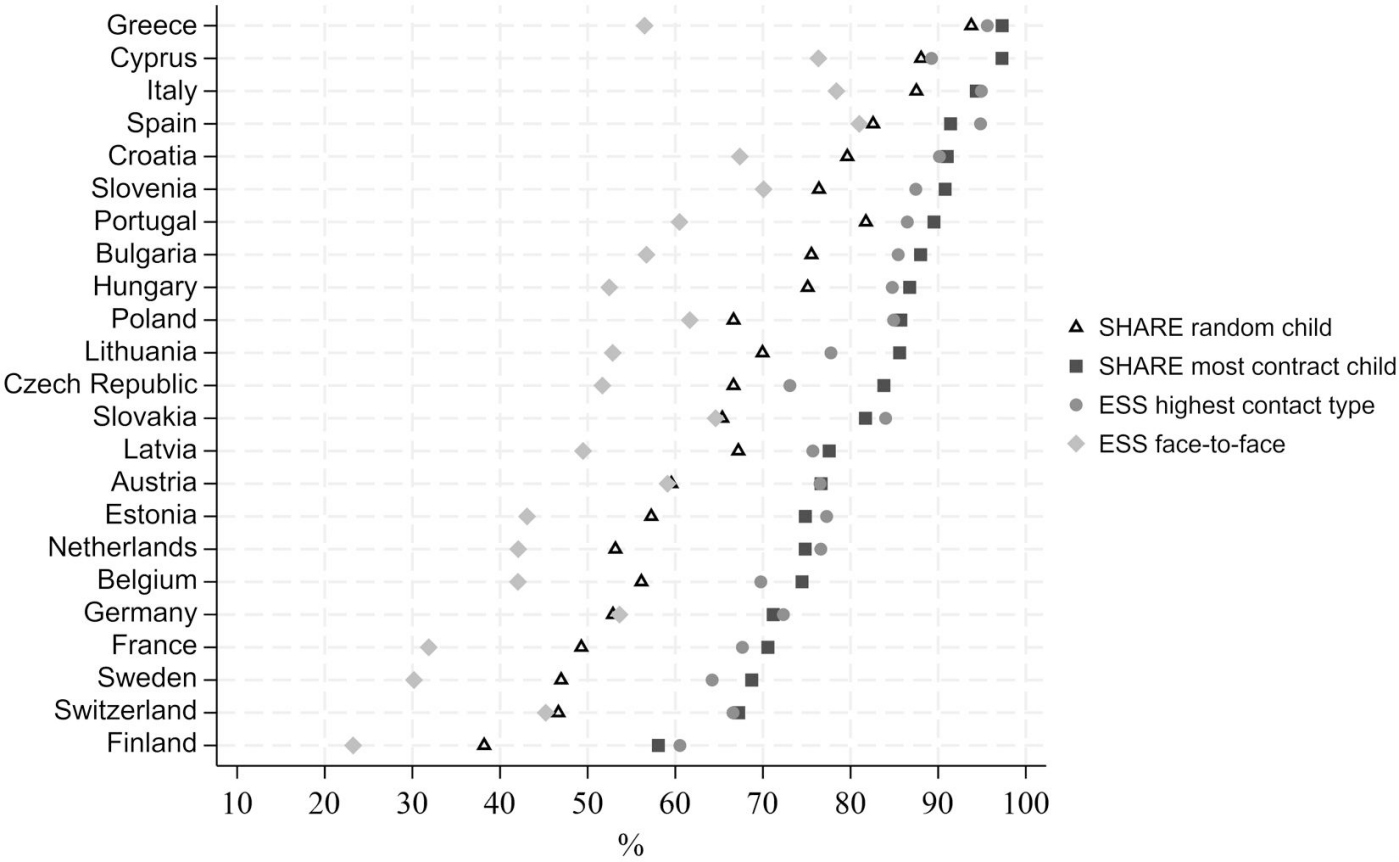
Prevalence of older adults (60+) having frequent (more than once a week) contact with children according to four contact measurement options. Countries are ordered according to the “SHARE most contacted child” measure. ESS = European Social Survey; SHARE = Survey of Health, Ageing and Retirement in Europe. *Source*: Survey of Health, Ageing and Retirement in Europe, wave 9 (SHARE-9; 2021–2022) and the European Social Survey, round 10 (ESS-10; 2020–2022).

Figure 1 displays frequent parent–child contact across countries using four measures: “SHARE most contacted child,” “ESS highest contact type,” “SHARE contact random child,” and “ESS face-to-face.” The numerical values are in Table S1. When ordering countries by “SHARE most contacted child,” the well-known North–South gradient emerges, where at least twice a week contact with at least one child is mostly common (around or above 90%) in Southern Europe (Greece, Italy, Spain, Portugal, and the “new” Southern cases Cyprus, Slovenia, Croatia), while Nordic and several Continental countries (e.g., Finland, Switzerland, Sweden, France) are at the bottom, often with around or below 70% reporting more than weekly contact. Eastern European countries occupy intermediate positions, with some (e.g., Hungary, Poland, Bulgaria) closer to Southern levels and other (e.g., Estonia) to Northern–Continental patterns.

Country rankings based on “SHARE most contacted child” and “ESS highest contact type” are extremely similar (ρ = 0.99; Figure 1, Table S2), with average levels differing by only about 2 percentage points, on average. This similarity is somewhat unexpected: random-child estimates on ESS should be lower than those based on the most-contacted child with SHARE. Moreover, one might expect “ESS highest contact type” to resemble “SHARE contact random child” more closely, as both refer to a random child. However, “SHARE contact random child” estimates are considerably lower (on average by 14 percentage points) than “ESS highest contact type” ones. For example, in Spain and Sweden, prevalence drops substantially when switching from the most-contacted to the random child approach. Nevertheless, ranks across the three measures remain highly correlated (ρ ≥ 0.94; Table S2), signaling robust cross-national contrasts despite substantial level differences.

The “ESS face-to-face” measure isolates frequent in-person contact with the random child and shows greater variability across and within regions. As expected, percentages are lower than for any mode of contact, but the ranking remains broadly similar. Southern countries again rank high, with Spain, Italy, and Cyprus showing the highest levels of frequent face-to-face contact (60%–80%), while Nordic countries and France show the lowest levels (23%–30%). Eastern and Continental countries fall in between. In Southern countries, characterized by high levels of overall frequency of contact, frequent face-to-face contact is particularly high. An exception is Greece, which ranks very high in overall frequency of contact but noticeably lower on face-to-face contact, indicating greater reliance on remote modes, mostly phone calls. Austria, Germany, and Switzerland occupy relatively higher positions on face-to-face than on any mode of contact, indicating that overall contact in these countries is comparatively more dependent on in-person meetings. However, the Spearman correlation coefficients of the “ESS face-to-face” measure with each of the other three measures remain relatively high (0.79–0.85; Table S2).

### Modes of contact

**Figure 2:**
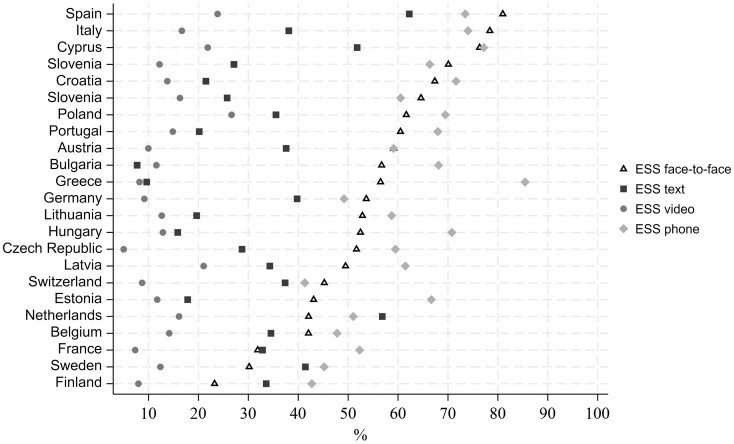
Prevalence of older adults (60+) having frequent contact (more than once a week) with children according to contact types in ESS data. Countries are ordered according to the “ESS face-to-face” measure. ESS = European Social Survey. *Source*: European Social Survey, round 10 (ESS-10; 2020–2022).

Figure 2 (Table S3) distinguishes four modes of frequent contact in ESS-10: face-to-face, phone, text, and video. Frequent phone contact broadly parallels face-to-face patterns (*ρ *= 0.75; Table S4). Southern countries show the highest levels of both, while in Greece, high phone contact partly compensates for more modest face-to-face contact. Nordic countries lie at the bottom; some Eastern countries (Bulgaria, Estonia, Hungary, Poland) reach levels similar to Southern Europe. The other Eastern and Continental countries usually lie in the middle. Bringing digital modes into the picture and distinguishing them from face-to-face and phone contact makes cross-national patterns of parent–child contact less straightforward. Frequent text messaging does not follow a simple regional gradient: Spain, the Netherlands, and Cyprus rank highest (>50%), followed by several Western/Nordic countries, while some Southern (Greece, Portugal) and Eastern (e.g., Bulgaria, Hungary) countries show much lower levels (<20%). The proportion of older parents having frequent video calls with children remains very low everywhere; even in the top countries, only a minority of parents engage more than once a week by using video calls, and in several countries, the share is below 10%. Low Spearman correlations for most pairs (negative for the phone-text pair) indicate that countries differ not only in how often parents and children are in touch, but also in how they keep in touch.

### Geographical proximity

**Figure 3:**
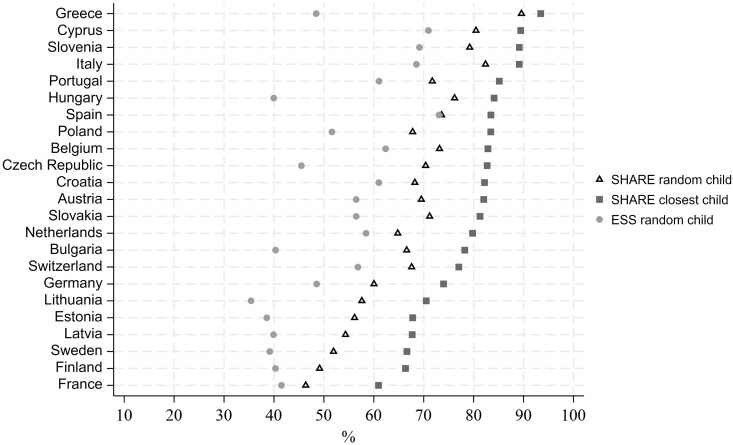
Prevalence of older adults (60+) living close (<25 km or <30 min) to children according to three different measures. Countries are ordered according to the “SHARE closest child” measure. ESS = European Social Survey; SHARE = Survey of Health, Ageing and Retirement in Europe. *Source*: Survey of Health, Ageing and Retirement in Europe, wave 9 (SHARE-9; 2021–2022) and the European Social Survey, round 10 (ESS-10; 2020–2022).

Figure 3 (Table S5) shows the share of parents living “close” to their children using three measures: “SHARE closest child” (at least one child living within 25 km), “SHARE close random child” (random child living within 25 km), and “ESS close random child” (random child living within 30 min). Ordered by “SHARE closest child,” we again observe a South–East–Continental–North gradient. The share of parents with at least one child within 25 km is very high (around 90%) in Southern countries (especially Greece, Cyprus, Italy), high (around 80%) in many Eastern and some Continental countries (e.g., Austria, Belgium), and lower (60%–65%) in Nordic countries and France. Switching to “SHARE random child” reduces prevalence by about 12 percentage points but largely preserves rank orders (*ρ* = 0.96; Table S6). Using the “ESS close random child” measure based on 30 min of travel time yields systematically lower prevalence than the “SHARE close random child” measure based on 25 km, with an average reduction of about 14 percentage points and a considerably lower rank-order correlation (*ρ* = 0.67). Differences are particularly pronounced for Greece and Hungary (downward shifts) and for Spain, the Netherlands, and Switzerland (upward shifts). Thus, the broad geographical gradient remains visible, but the moderate cross-survey correlation indicates that proximity rankings are not interchangeable across measures of spatial distance and travel time.

### Robustness checks

We implemented three sets of robustness checks (results available upon request). First, we re-estimated all indicators of frequent contact and proximity using regression models adjusting for age, age squared, gender, number of children, and partnership status, and derived marginal country-level probabilities. Adjusted and unadjusted estimates were very similar, and rank-order correlations were consistently very high (typically >0.95). This suggests that institutional and cultural factors, rather than compositional differences, are important for understanding the observed patterns. Second, we examined the effect of varying proximity thresholds. Our baseline defined “living close” as within 25 km (SHARE) and within 30 min (ESS). Because the 25 km threshold proved less restrictive than 30 min and some country rankings differed substantially, we recalculated prevalence using a 5 km threshold in SHARE and a 45-min threshold in ESS, always for the random child. Changing thresholds strongly affects prevalence but leaves rankings within each survey highly stable (*ρ* = 0.95 for ESS, *ρ* = 0.88 for SHARE). Cross-survey concordance remains stable but relatively weak (*ρ *≈ 0.67–0.72). Third, we examined the role of co-residence, given that treating co-resident dyads as having frequent contact may partly capture structural living arrangements rather than active contact maintenance, which is always required across separate households. We therefore recalculated the contact indicators after excluding co-resident parent–child dyads. As expected, excluding co-resident dyads lowers prevalence levels, especially for face-to-face contact and in Southern European countries. However, country rankings remain highly stable. For the four measures shown in Figure 1, Spearman correlations between estimates with and without co-residence range from 0.979 to 0.996, while average differences in prevalence range from 2.52 to 6.45 percentage points. For the mode-specific ESS indicators, the effect is concentrated almost entirely in face-to-face contact: the corresponding rank-order correlations range from 0.979 to 0.991, and the average difference is 6.45 percentage points for face-to-face contact but close to zero for phone, text, and video contact. These results suggest that co-residence affects prevalence levels more than the cross-national ordering of countries.

## Discussion

### Updated cross-national evidence on older parents–children contact and proximity

This study first provides an updated portrait of contact and geographical proximity between older parents and their adult children across Europe. Using recent SHARE and ESS data, we confirm a well-known but still striking pattern: frequent intergenerational contact (Figure 1) and close residential proximity (Figure 3) are most prevalent in Southern Europe and least common in Nordic and several Continental countries, even after adjusting for basic socio-demographic factors and excluding co-residence. This aligns with longstanding distinctions between “strong” and “weak” family regimes (Reher, 1998; Saraceno & Keck, 2010) and with work on later-life family typologies and intergenerational solidarity in Europe (Dykstra & Fokkema, 2011; Hank, 2007; Tomassini et al., 2004). By incorporating additional Southern and Eastern European countries, we show that the “new” Southern cases, such as Cyprus, largely reinforce the strong-family pattern, whereas Eastern European countries tend to occupy intermediate positions between Southern and Nordic contexts, with substantial internal heterogeneity. Balkan countries, particularly Slovenia and Croatia, tend to have high levels of contact similar to Southern countries. Baltic European countries, particularly Estonia and Latvia, show levels of contact more similar to Central Western and Nordic countries. This suggests a gradient of intergenerational cohesion across Europe, rather than a simple North–South or East–West dichotomy. These results are consistent with interpretations that emphasize welfare regimes, housing markets, family norms, and post-socialist legacies as relevant contextual mechanisms (Castiglioni et al., 2016; Herlofson et al., 2011; Saraceno & Keck, 2010), even though testing such macro-level explanations is beyond the scope of the present descriptive and methodological analysis and can be the objective of future research.

### Cross-national variations in digital contact

A second key substantive contribution is the systematic comparative analysis of digital parent–child contact (Figure 2). Conceptually, digital communication could be substitutive if it displaced face-to-face or telephone interaction, or supplementary if it added channels of connection alongside them. Our findings are more consistent with the latter interpretation at the country level. Frequent face-to-face meetings and phone calls remain the backbone of parent–child ties, while text messaging is less widespread and frequent video calls are still relatively rare. This pattern is consistent with evidence that digital communication among older adults tends to cumulate with, rather than substitute for, offline interaction (Arpino & Failli, 2024b; Danielsbacka et al., 2023; Tosi & Arpino, 2025) and with the notion of “digital solidarity,” whereby technology helps maintain relationships when physical encounters are constrained (Hwang, 2026; Hwang et al., 2024). However, we do find some exceptions in low digitalized countries (e.g., Bulgaria and Portugal) where high levels of frequent face-to-face and phone contact are found together with low levels of digital contact (video and text messages).

Our results contribute to the intergenerational solidarity literature (Bengtson & Roberts, 1991) by showing that digital contact can be understood not as a wholly separate dimension of solidarity, but as a new channel through which associational solidarity may be enacted. At the same time, the uneven diffusion of digital contact across and within countries points to possible new forms of inequality in intergenerational connectedness. Some older parents may at the same time have children living at a distance and low digital skills, which may imply a cumulative disadvantage of low face-to-face and digital solidarity. Future research should examine whether limited face-to-face contact and limited ability to use digital communication accumulate among some sub-population groups, and with what implications for wellbeing.

### The role of measurement choices

Third, this study shows that in some cases, measurement choices shape the conclusions we draw about cross-national patterns in parent–child relationships. Methodologically, we in fact contribute to prior literature by systematically comparing indicators based on various operationalization choices. Relying on the “most contacted child” systematically gives a higher prevalence of intensive contact compared with using a random child approach, yet the relative position of countries remains remarkably stable (Figure 1). This stability, however, should not be over-interpreted: it partly reflects broad regional gradients and does not erase sizeable differences in prevalence levels. The contrast is sharper for geographical proximity. Thresholds in different metrics (e.g., 25 km vs. 30 min) substantially affect prevalence levels, and the correspondence between the SHARE and ESS random-child proximity rankings is relatively weak (Figure 3). Countries such as Greece and Hungary illustrate how geography, transport infrastructure, and settlement patterns can produce short average physical distances but longer travel times. For comparative researchers, the implication is that measurement choices can affect not only levels but also some substantive cross-national conclusions. We thus caution against taking any single distance cutoff as definitive evidence of “close-knit” families without considering context. At a theoretical level, this touches on the *structural* dimension of intergenerational solidarity: physical distance is a key structural constraint/enabler, but its impact is moderated by infrastructure and policy (e.g., availability of affordable housing near kin, transport networks, urban versus rural settlement patterns). Our Greek example suggests that some apparent regional differences in family proximity might be exacerbated or alleviated by such contextual factors, which merits further investigation. More generally, it is worth noting that the comparison between the tested most-contacted and random-child indicators also reflects a substantive distinction: the former captures access to at least one frequently contacted child, whereas the latter captures contact with a typical child. Random-child items can therefore be useful when the goal is to estimate aggregate average-child patterns in large general surveys with limited questionnaire space. They are less appropriate when the research question concerns within-family heterogeneity, selectivity across children, sibling interdependence, or whether parents have at least one frequently contacted or nearby child.

### Limitations

Our study also has limitations. First, we provide cross-sectional snapshots, rather than analyzing how proximity and contact modes evolve over the life course (Choi et al., 2021) or in response to shocks such as the COVID-19 pandemic (Vergauwen et al., 2022). Second, we rely on parents’ reports and frequency-based indicators, without information on the quality, emotional tone, or content of contact. Frequent contact can be supportive or conflictual, with very different implications for wellbeing (Connidis & Barnett, 2018; Fingerman et al., 2020). Third, our proximity and contact measures are necessarily coarse: thresholds (25 km/30–45 min) and broad contact categories (phone, text, video) cannot capture the full diversity of communication practices or travel conditions. Future work should use longitudinal and dyadic designs linking parents and multiple children, incorporate time-use or diary data, and collect more granular information on multi-modal communication and continuous distance and travel-time measures.

## Conclusions

Despite these limitations, our study offers a timely and nuanced view of intergenerational relations in an aging, increasingly digital Europe. Substantively, we show that strong regional patterns in proximity and contact persist, while digital channels are becoming an important, but still limited, complement to traditional forms of intergenerational solidarity. Methodologically, we demonstrate that conclusions may partially depend on how parent–child ties are operationalized, and that some cross-national orderings are more robust than others. For policy, our findings underscore the need for an integrated approach that supports both physical and digital connectedness: housing and transport policies that facilitate living near or visiting kin, and investments in age-friendly digital infrastructure and literacy. Because where older parents and their adult children live in relation to one another, and how they stay in touch, shape everyday opportunities for care, practical help, and emotional support, mapping the patterns in parent–child proximity and contact is essential for understanding inequalities in later-life health and wellbeing and should remain a central concern for gerontological research and aging policy.

## Supplementary Material

gbag117_Supplementary_Data

## Data Availability

We use secondary data from: (a) the Survey of Health and Retirement in Europe (SHARE) that can be obtained at: share-eric.eu/data/; and (b) the European Social Survey (ESS) that can be obtained at: https://ess.sikt.no/en/.
