# Supplementary material for: Older parent–adult child contact and proximity across Europe: new evidence, digital communication, and measurement issues
Source: J Gerontol B Psychol Sci Soc Sci. 2026 Jun 27;81(9):gbag117. doi: 10.1093/geronb/gbag117 (PMC13476080; doi:10.1093/geronb/gbag117)
Supplement: Supplementary file 1 [file gbag117_supplementary_data.pdf]

The Journals of Gerontology, Series B: Psychological Sciences and Social Sciences

Supplementary Material: Arpino, Tosi, & Bordone. Older parent–adult child contact and proximity across Europe: New evidence, digital communication, and measurement issues.

Supplementary Table 1. Prevalence of older adults (60+) having frequent (more than once a week) contact with children according to four contact measurement options. (Numerical values corresponding to estimates in Figure 1)

	(1)	(2)	(3)	(4)
**Country**	**SHARE contact random child**	**SHARE most contacted child**	**ESS highest contact type**	**ESS face-to-face**
Greece	0.9378	0.9730	0.9561	0.5650
Cyprus	0.8806	0.9729	0.8924	0.7633
Italy	0.8751	0.9435	0.9493	0.7839
Spain	0.8258	0.9142	0.9483	0.8100
Croatia	0.7963	0.9104	0.9014	0.6736
Slovenia	0.7640	0.9080	0.8745	0.7008
Portugal	0.8176	0.8953	0.8648	0.6048
Bulgaria	0.7554	0.8800	0.8544	0.5671
Hungary	0.7511	0.8675	0.8477	0.5247
Poland	0.6665	0.8574	0.8492	0.6165
Lithuania	0.6995	0.8561	0.7776	0.5286
Czech Republic	0.6665	0.8380	0.7308	0.5167
Slovakia	0.6536	0.8171	0.8401	0.6460
Latvia	0.6720	0.7757	0.7570	0.4948
Austria	0.5955	0.7664	0.7652	0.5911
Estonia	0.5725	0.7484	0.7727	0.4309
Netherlands	0.5317	0.7483	0.7661	0.4208
Belgium	0.5613	0.7448	0.6976	0.4205
Germany	0.5289	0.7119	0.7233	0.5363
France	0.4928	0.7057	0.6766	0.3187
Sweden	0.4697	0.6873	0.6420	0.3018
Switzerland	0.4666	0.6725	0.6659	0.4522
Finland	0.3819	0.5807	0.6053	0.2324

Sources: Survey of Health, Ageing and Retirement in Europe, wave 9 (SHARE-9; 2021–2022) and the European Social Survey, round 10 (ESS-10; 2020–2022).

Supplementary Table 2. Spearman correlation coefficients among the country rankings based on the four measures of frequent parent-child contact displayed in Figure 1.

	**ESS face-to-face**	**ESS highest contact**	**SHARE most contacted child**	**SHARE contact random child**
**ESS face-to-face**	1			
**ESS highest contact**	0.85	1		
**SHARE most contacted child**	0.83	0.97	1	
**SHARE contact random child**	0.79	0.94	0.99	1

Sources: Survey of Health, Ageing and Retirement in Europe, wave 9 (SHARE-9; 2021–2022) and the European Social Survey, round 10 (ESS-10; 2020–2022).

Supplementary Table 3. Prevalence of older adults (60+) having frequent contact (more than once a week) with children according to four contact types in ESS data. (Numerical values corresponding to estimates in Figure 2)

Country	(1)	(2)	(3)	(4)
	**ESS face-to-face**	**ESS phone**	**ESS text**	**ESS video**
Spain	0.8100	0.7346	0.6226	0.2384
Italy	0.7839	0.7402	0.3811	0.1669
Cyprus	0.7633	0.7719	0.5181	0.2186
Slovenia	0.7008	0.6635	0.2712	0.1221
Croatia	0.6736	0.7162	0.2149	0.1376
Slovakia	0.6460	0.6051	0.2575	0.1630
Poland	0.6165	0.6948	0.3554	0.2663
Portugal	0.6048	0.6798	0.2017	0.1485
Austria	0.5911	0.5910	0.3758	0.0997
Bulgaria	0.5671	0.6813	0.0772	0.1158
Greece	0.5650	0.8545	0.0965	0.0819
Germany	0.5363	0.4918	0.3977	0.0917
Lithuania	0.5286	0.5873	0.1964	0.1265
Hungary	0.5247	0.7079	0.1586	0.1288
Czech Republic	0.5167	0.5948	0.2873	0.0503
Latvia	0.4948	0.6146	0.3431	0.2105
Switzerland	0.4522	0.4132	0.3737	0.0871
Estonia	0.4309	0.6668	0.1785	0.1174
Netherlands	0.4208	0.5103	0.5685	0.1612
Belgium	0.4205	0.4780	0.3453	0.1414
France	0.3187	0.5229	0.3285	0.0733
Sweden	0.3018	0.4519	0.4144	0.1238
Finland	0.2324	0.4272	0.3359	0.0797

Source: European Social Survey, round 10 (ESS-10; 2020–2022).

Supplementary Table 4. Spearman correlation coefficients among the country rankings based on the four measures of frequent parent-child contact displayed in Figure 2.

	**ESS face-to-face**	**ESS phone**	**ESS text**	**ESS video**
**ESS face-to-face**	1			
**ESS phone**	0.75	1		
**ESS text**	0.05	-0.25	1	
**ESS video**	0.52	0.45	0.34	1

Source: European Social Survey, round 10 (ESS-10; 2020–2022).

Supplementary Table 5. Prevalence of older adults (60+) living close (<25km or < 30min) to children according to three different measures. (Numerical values corresponding to estimates in Figure 3)

	(1)	(2)	(3)
**Country**	**SHARE close random child**	**SHARE closest child**	**ESS close random child**
Greece	0.8958	0.9344	0.4845
Cyprus	0.8047	0.8942	0.7094
Slovenia	0.7921	0.8915	0.6914
Italy	0.8238	0.8913	0.6853
Portugal	0.7171	0.8514	0.6105
Hungary	0.7618	0.8410	0.3996
Spain	0.7358	0.8346	0.7305
Poland	0.6777	0.8344	0.5161
Belgium	0.7316	0.8286	0.6237
Czech Republic	0.7039	0.8270	0.4550
Croatia	0.6822	0.8219	0.6101
Austria	0.6952	0.8202	0.5647
Slovakia	0.7119	0.8128	0.5647
Netherlands	0.6480	0.7979	0.5845
Bulgaria	0.6658	0.7822	0.4032
Switzerland	0.6758	0.7703	0.5681
Germany	0.6003	0.7397	0.4855
Lithuania	0.5760	0.7051	0.3542
Estonia	0.5615	0.6779	0.3856
Latvia	0.5433	0.6770	0.3991
Sweden	0.5193	0.6663	0.3915
Finland	0.4914	0.6636	0.4031
France	0.4636	0.6095	0.4151

Sources: Survey of Health, Ageing and Retirement in Europe, wave 9 (SHARE-9; 2021–2022) and the European Social Survey, round 10 (ESS-10; 2020–2022).

Supplementary Table 6. Spearman correlation coefficients among the country rankings based on the three measures of parent-child proximity displayed in Figure 3.

	**ESS close random child**	**SHARE closest child**	**SHARE close random child**
**ESS close random child**	1		
**SHARE closest child**	0.674	1	
**SHARE close random child**	0.671	0.964	1

Sources: Survey of Health, Ageing and Retirement in Europe, wave 9 (SHARE-9; 2021–2022) and the European Social Survey, round 10 (ESS-10; 2020–2022).
